# Acute limb ischemia in patients with COVID-19 pneumonia

**DOI:** 10.1016/j.amsu.2021.102747

**Published:** 2021-08-25

**Authors:** Amine Bensaid, Imane Melhaoui, Younes Oujidi, Abdelilah El Rhalete, Inass Arhoun El Haddad, Houssam Bkiyar, Brahim Housni

**Affiliations:** a Intensive Care Unit, Mohammed VI University Hospital, Faculty of Medicine and Pharmacy, Oujda, Morocco; b Radiology Department, Mohammed VI University Hospital, Faculty of Medicine and Pharmacy, Oujda, Morocco

**Keywords:** Acute limb ischemia, Arterial thrombosis, COVID-19, Severe acute respiratory syndrome coronavirus 2

## Abstract

The severe acute respiratory syndrome coronavirus 2 (SARS-CoV-2) pandemic developing since the late 2019 and early 2020 has caused thousands of deaths and has had an enormous impact on our health systems and economies. Thrombotic complications associated with coronavirus disease 2019 (COVID-19) have been described. Acute limb ischemia is associated with complications related to cytokine storm syndrome and acute respiratory distress syndrome. that can result in significant morbidity and mortality. However, limited published data is available regarding thrombosis in coronavirus disease 2019 (COVID-19). Here we are presenting 2 cases of COVID-19 infection complicated by arterial thrombosis in the form of acute limb ischemia.

## Highlights

• COVID-19 pneumonia increases the incidence of acute limb ischemia.
•Thrombotic complications associated with coronavirus disease 2019 can result in significant morbidity and mortality.
•Patients with COVID-19 should receive pharmacologic thromboprophylaxis.

## 1 Introduction

At the end of 2019, a novel coronavirus was identified as the cause of a cluster of pneumonia cases in Wuhan, a city in the Hubei Province of China. On March 11, 2020, WHO declared coronavirus disease 2019 (COVID-19) as pandemic [1].

The association of COVID-19 with coagulopathy has gained increasing interest recently. As per American Society of Hematology, some patients with severe coronavirus disease 2019 (COVID-19) have fulminant activation of coagulation and consumption of coagulation factors, which meets the criteria for disseminated intravascular coagulation as per International Society on Thrombosis and Hemostasis [2]. A meta-analysis was conducted by Xiong et al. [3] which indicated that prothrombin time and D-dimer levels were significantly higher in patients with severe COVID-19 than in those with the mild disease. Our study has been reported in line with the SCARE 2020 criteria [16].

## 2 Case presentation

### 2.1 Case 1

80 years old patient, diabetic, hypertensive, presented to emergency department with complaints of shortness of breath and chest pain, with a discoloration and swelling of the right leg. Vital signs upon arrival were: heart rate at 113 per minute, blood pressure at 140/85 mmHg, oxygen saturation at 40% on room air and temperature at 38,6° Celsius.

**Fig. 1:**
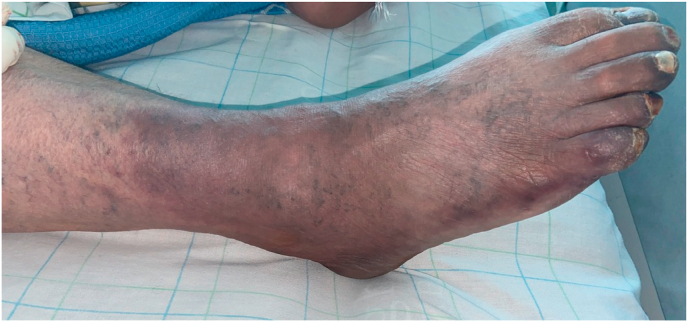
Cyanotic right foot showing the acute limb ischemia.

The patient had bilateral crackles on lung exam and absent right popliteal, dorsalis pedis and posterior tibial pulses and the foot was swollen, cyanotic, and cold [Fig. 1].

On initial laboratory evaluation, the following values were noted: hemoglobin 11.1 g/dl (reference: 12–16 g/dl), hematocrit 33,6% (reference: 36%–46%), white blood cells 27.680/mm^3^ (reference: 4.000–10.000/mm^3^), platelets 239.000/mm^3^ (reference: 150.000–400.000/mm^3^), potassium 5.1 meq/L (reference: 3.5–5,5 meq/L), glucose 3,35g/l (reference: 0,7–1,10 g/l), creatinine 26,2 mg/l (reference: 6–13 mg/l), blood urea nitrogen 1,6 g/l (reference: <0,5 g/l), lactic acid 2,37 mmol/L (reference: 0.5–2.2 mmol/L), troponin 1150 ng/l (reference: less than 6 ng/l), D-dimer 35,2 (reference: less than 0.5), prothrombin time 67,% (reference: 70–100%, international normalized ratio (INR) 1.24 (reference: less than 1), Creatine Phosphokinase (CPK) 2080 U/L (reference; 10–200 U/l) lactate dehydrogenase (LDH) 1497 U/L (reference: 140–271 U/L), C-reactive protein (CRP) 239 mg/L (reference: less than 10 mg/L), ferritin 1642 ng/L (reference: 12–300 ng/l), procalcitonin 2,88 ng/ml (reference: less than 2 ng/ml), calcium 9,1 mg/dl (reference: 8.6–10.3 mg/dl), and albumin 29 mg/l (reference: 35–50 mg/l).

**Fig. 2:**
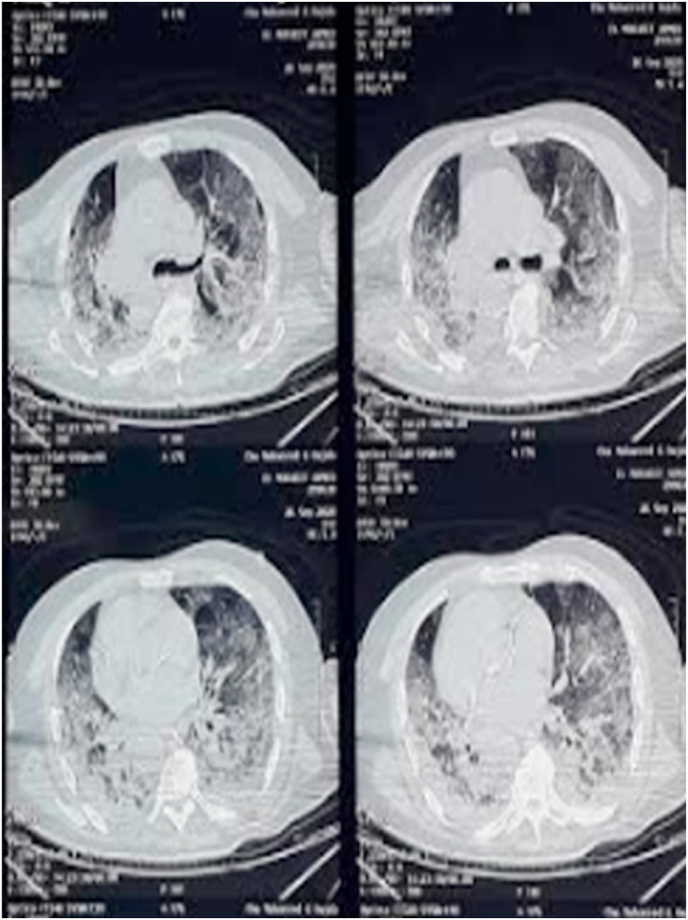
Pre-operative computed tomography scan of an 80 years old man with COVID-19- related pneumonia and acute limb ischemia. Transverse thin section scans show extensive ground-glass opacities of both lungs.

**Fig. 3:**
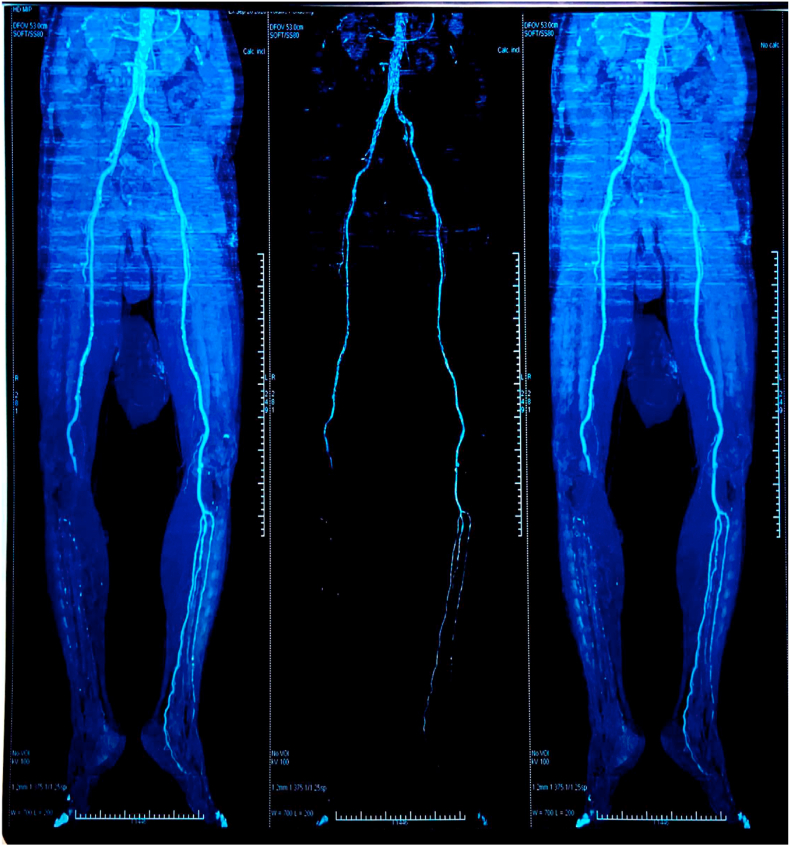
Pre-operative computed tomography angiography with 3-Dimensional reconstruction showing the presence of intraluminal thrombus at iliofemoral artery, total occlusion of popliteal segment, and the absence of flow below.

Chest X ray showed bilateral hazy infiltrates [Fig. 2]. Thoracic and Abdominal CT angiography of the aorta with Iliofemoral runoff showed atheroma plaque within the right popliteal artery and occluded right anterior tibial artery, right posterior tibial artery, and right peroneal artery with no flow to the foot [Fig. 3].

Covid-19 was diagnosed on the basis of reverse transcription polymerase chain reaction (RT- PCR) testing. Echocardiogram showed an ejection fraction of 55%. The patient was intubated and put under SDRA ventilation and was started on moxifloxacin, ceftriaxone, azithromycin, hydroxychloroquine, and therapeutic anticoagulation with heparin. He underwent a thigh amputation.

### 2.2 Case 2

69 years old patient, suffering from chronic obstructive pulmonary disease, presented to emergency department with complaints of shortness of breath and chest pain, with a discoloration and swelling of the left leg. Vital signs upon arrival were: heart rate at 130 per minute, blood pressure at 150/95 mmHg, oxygen saturation at 75% on room air and temperature at 38,1° Celsius.

The patient had bilateral crackles on lung exam and absent left popliteal, dorsalis pedis and posterior tibial pulses and the foot was swollen, and cold.

Electrocardiogram showed sinus tachycardia 140 per minute.

On initial laboratory evaluation, the following values were noted: hemoglobin 15.1 g/dl (reference: 12–16 g/dl), hematocrit 42,5% (reference: 36%–46%), white blood cells 21.560/mm^3^ (reference: 4.000–10.000/mm^3^), platelets 345.000/mm^3^ (reference: 150.000–400.000/mm^3^), potassium 4,8 meq/L (reference: 3.5–5,5 meq/L), glucose 1,16 g/l (reference: 0,7–1,10 g/l), creatinine 7,25 mg/l (reference: 6–13 mg/l), blood urea nitrogen 0,56 g/l (reference: <0,5 g/l), lactic acid 2,37 mmol/L (reference: 0.5–2.2 mmol/L), troponin 9,5 ng/l (reference: less than 6 ng/l), D-dimer 12,96 (reference: less than 0.5), prothrombin time 78% (reference: 70–100%, international normalized ratio (INR) 1.13 (reference: less than 1), Creatine Phosphokinase (CPK) 680 U/L (reference; 10–200 U/l) lactate dehydrogenase (LDH) 1040 U/L (reference: 140–271 U/L), C-reactive protein (CRP) 269 mg/L (reference: less than 10 mg/L), ferritin 854 ng/L (reference: 12–300 ng/l), procalcitonin 0,51 ng/ml (reference: less than 2 ng/ml), calcium 8,3 mg/dl (reference: 8.6–10.3 mg/dl), and albumin 33 mg/l (reference: 35–50 mg/l).

**Fig. 4:**
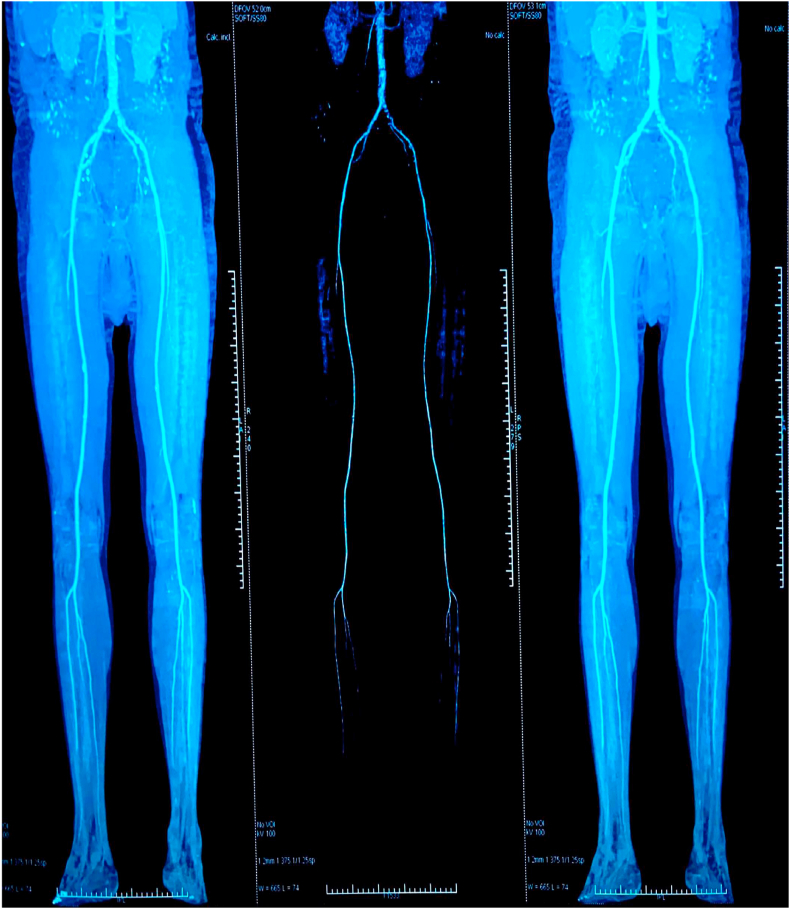
Pre-operative computed tomography angiography with 3-Dimensional reconstruction showing the presence of intraluminal thrombus at iliofemoral artery, total occlusion of popliteal segment, and the absence of flow below.

Chest X ray showed bilateral hazy infiltrates. Thoracic and Abdominal CT angiography of the aorta with Iliofemoral runoff showed an occluded left anterior tibial artery and left peroneal artery with no flow to the foot [Fig. 4].

**Fig. 5:**
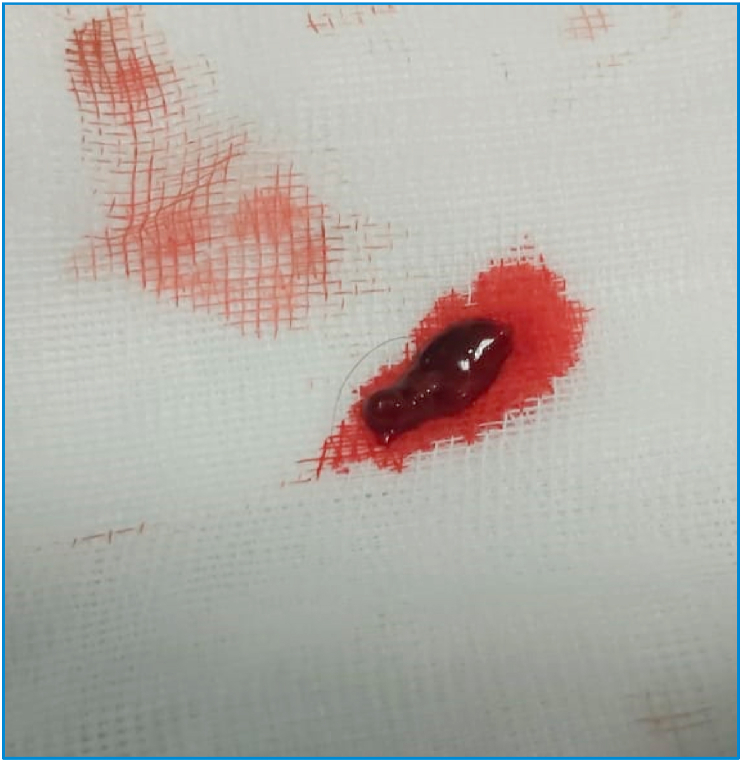
Surgical specimen of the thrombectomy.

Covid-19 was diagnosed on the basis of reverse transcription polymerase chain reaction (RT- PCR) testing. Echocardiogram showed an ejection fraction of 60%. The patient was admitted to intensive care unit and put under high flow nasal oxygenation (Optiflow) and was started on azithromycin, hydroxychloroquine, and therapeutic anticoagulation with heparin. He underwent a thrombectomy of left common femoral artery, profunda femoris, superficial femoral artery, popliteal artery, anterior tibial artery, posterior tibial artery, and peroneal artery [Fig. 5].

## 3 Discussion

The COVID-19 pandemic is a fast-evolving situation. The spectrum of clinical manifestations of SARS-CoV-2 infection includes fever, myalgia, cough and dyspnea, and less frequently headache, diarrhea, nausea and vomiting [4]. Severity of infection could be varied from asymptomatic infection to critical disease. Smell and taste disorders (e.g., anosmia and dysgeusia) have also been reported as common symptoms in patients with COVID-19 [5]. Although in COVID-19 respiratory symptoms predominate, thrombosis can occur with COVID-19 [6]. COVID-19 is frequently severe in patients with advanced age and medical comorbidities (cardiovascular disease, diabetes mellitus, hypertension, chronic lung disease, cancer, chronic kidney disease, obesity, and smoking) [7].

The hypercoagulability of critically ill patients with COVID-19 became evident. In addition, some published epidemiologic data have shown increased thromboembolic events in those patients, such as unusual ischemic limbs and venous thromboembolism [8,9]. A high D-dimer levels, high fibrinogen levels, and low antithrombin levels has been observed in hospitalized Covid-19 patients who developed thromboembolic events [10]. Moreover, Tang et al. [11] in their study found that 74% of patients who died of COVID-19 had disseminated intravascular coagulopathy.

Cui et al. [12] in a retrospective study analyzed 81 patients with severe COVID-19 in the intensive care unit (ICU). The incidence of venous thromboembolism (VTE) in these patients was 25% (20/81) and may be related to poor prognosis. Furthermore, Klok et al. [13] analyzed 184 patients with COVID-19 in ICU; the incidence of thrombosis was 31%; 27% were VTE and 3.7% were arterial thrombotic events. Our case adds to the literature that life-threatening acute limb ischemia can occur in COVID-19 patients.

Acute limb ischemia is a vascular emergency and can result in severe morbidity, including chronic pain, limb loss, and severe disability. A prompt diagnosis is a prerequisite for successful treatment. An observational study done in Italy revealed increased incidence of acute limb ischemia in patients with COVID-19 infections, they have noted the number of patients with acute limb ischemia during the peak of the pandemic has increased [14].

The mechanism of thromboembolic complications associated with COVID-19 infection is not fully understood. COVID-19 related hypercoagulability is likely multifactorial-direct viral infection of the endothelial cell leading to diffuse endothelial inflammation, increased procoagulant factors such as factor VIII, von Willebrand factor, fibrinogen, and high inflammatory state associated with the cytokine storm leading to coagulation and fibrinolysis activation [15].

The American Society of Hematology (ASH) recommends that all hospitalized patients with COVID-19 should receive pharmacologic thromboprophylaxis with LMWH or fondaparinux, unless bleeding risk exists, and full therapeutic-intensity anticoagulation in the appropriate clinical scenario [11].

## 4 Conclusions

We report 2 cases of arterial thrombosis in patients with COVID-19. Our case and review of literature reveals that health care providers should be aware of this unusual life-threatening manifestation of COVID 19 so that appropriate measures can be taken for the vascular emergency.

## Consent

Written informed consent was obtained from the patient for publication of this case report and any accompanying images.

## Ethical approval

This is a case report.

## Sources of funding

This study was not funded.

## Author contribution

Amine Bensaid: Stady concept, data collection, Data analysis, writing the paper. Imane Melhaoui: writing the paper. Oujidi Younes: Contributor. Inass Arhoun El Haddad: Data collection. Houssam Bkiyar: Supervision and data validation. Brahim Housni: Supervision and data validation.

## Guarantor

DR Amine Bensaid.

## Provenance and peer review

Not commissioned, externally peer reviewed.

## Declaration of competing interest

No conflict of interest.
